# Associations of metabolic syndrome, its severity with cognitive impairment among hemodialysis patients

**DOI:** 10.1186/s13098-023-01080-3

**Published:** 2023-05-23

**Authors:** Yuqi Yang, Qian Li, Yanjun Long, Jing Yuan, Yan Zha

**Affiliations:** 1 Deparment of Nephrology, Guizhou Provincial People’s Hospital, Guiyang, 550002 China; 2 NHC Key Laboratory of Pulmonary Immune-Related Diseases, Guizhou Provincial People’s Hospital, Guiyang, 550002 China

**Keywords:** Metabolic syndrome, Cognitive impairment, Hemodialysis, Severity, Dose–response

## Abstract

**Background:**

In the general population, metabolic syndrome (MetS) is associated with increased risk of cognitive impairment, including global and specific cognitive domains. These associations are not well studied in patients undergoing hemodialysis and were the focus of the current investigation.

**Methods:**

In this multicenter cross-sectional study, 5492 adult hemodialysis patients (3351 men; mean age: 54.4 ± 15.2 years) treated in twenty-two dialysis centers of Guizhou, China were included. The Mini-Mental State Examination (MMSE) was utilized to assess mild cognitive impairment (MCI). MetS was diagnosed with abdominal obesity, hypertension, hyperglycemia, and dyslipidemia. Multivariate logistic and linear regression models were used to examine the associations of MetS, its components, and metabolic scores with the risk of MCI. Restricted cubic spline analyses were performed to explore the dose–response associations.

**Results:**

Hemodialysis patients had a high prevalence of MetS (62.3%) and MCI (34.3%). MetS was positively associated with MCI risk with adjusted ORs of 1.22 [95% confidence interval (CI) 1.08–1.37, *P* = 0.001]. Compared to no MetS, adjusted ORs for MCI were 2.03 (95% CI 1.04–3.98) for 22.51 (95% CI 1.28–4.90) for 3, 2.35 (95% CI 1.20–4.62) for 4, and 2.94 (95% CI 1.48–5.84) for 5 components. Metabolic syndrome score, cardiometabolic index, and metabolic syndrome severity score were associated with increased risk of MCI. Further analysis showed that MetS was negatively associated with MMSE score, orientation, registration, recall and language (*P* < 0.05). Significant interaction effect of sex (*P* for interaction = 0.012) on the MetS-MCI was observed.

**Conclusion:**

Metabolic syndrome was associated with MCI in hemodialysis patients in a positive dose–response effect.

**Supplementary Information:**

The online version contains supplementary material available at 10.1186/s13098-023-01080-3.

## Background

Cognitive impairment is a common and critical health issue in patients with end-stage kidney disease (ESKD) receiving hemodialysis, the prevalence of different extents of cognitive impairment ranged from 70–80% [1–3]. The severity of cognitive impairment has a graded association with adverse outcomes, including functional impairment, quality of life, even dialysis withdrawal, and mortality [4–6]. Mild cognitive impairment (MCI) represents a transitional stage between normal age-related decline in cognitive function and dementia and is more prevalent in hemodialysis than the general population [7]. Previous studies have showed that MCI is positively associated with a high risk of progressing to dementia, and is still more likely to be improved or maintained cognitive function, compared to dementia [8]. The latest guideline recommends MCI as the proper therapeutic window [8], highlighting the importance of screening modifiable risk factors for early identification and targeted interventions to delay onset and progression.

Metabolic disturbance can cause a direct insult to endothelium and smooth muscle of the cerebral vasculature, which leads to cerebral vasoconstriction and hypoperfusion, and also disrupt key hemostatic processes in the brain, eventually contributing to cognitive deficits [9]. Metabolic syndrome (MetS) is a cluster of metabolic disturbances, including abdominal obesity, hypertension, hyperglycemia, and atherogenic dyslipidemia (increased triglycerides, and decreased high-density lipoprotein cholesterol levels) [10]. Current evidence suggests that one quarter of the world population suffer from MetS [11], which is much higher in hemodialysis patients, with the prevalence of 40–74.5% [12, 13]. As a constellation of cardiovascular risk factors, MetS has been proven to increase the risk of cardiovascular disease, mortality and other adverse health outcomes in hemodialysis patients [14, 15]. More importantly, there is growing evidence suggesting an association between MetS and ischemia brain changes, accelerated cognitive decline, cognitive impairment and dementia [16–20]. Recent studies have demonstrated that MetS is a risk factor for cognitive impairment among general population [16], no matter young [17], middle-aged [18] or elderly individuals [19, 20]. In addition, several novel metabolic indices, such as metabolic syndrome (siMS) score [21], cardiometabolic index (CMI) [22] and metabolic syndrome severity score (MetSSS) [23], have been demonstrated to be promising indicators for predicting and quantifying MetS and its severity, with relatively high sensitivity and specificity. However, these associations between these indices and MCI are yet unknown.

Considering the high prevalence of MetS and cognitive impairment in hemodialysis patients, we suspected a potential association between these two critical complications. However, few studies have been reported among the specific population. The aim of this study was to investigate the associations of MetS, its components and severity scores with MCI among hemodialysis patients.

## Methods

### Ethics statement

This study followed the Declaration of Helsinki, and was reviewed and approved by the Institutional Review Board of Guizhou Provincial People’s Hospital [Approval Number: (2020)208]. All participants provided written informed consent.

### Study design and participants

This multicenter, cross-sectional study included patients who undergo maintenance hemodialysis in twenty-two dialysis centers of Guizhou Province, China between June 2019 and September 2021. All the patients performed hemodialysis, a process in which blood is drained outside the body through a circulatory line, exchanged through a dialyzer composed of essential electrolyte concentrations under the standard temperature (35.5–36.5 °C), and the purified blood is returned to the body, with the proper vascular access, such as fistulas, and catheters. Participants aged  ≥ 18 years old, undergoing hemodialysis for at least three months, twice or trice per week, and those completed biochemical, anthropometric measurements and questionnaire records were included. Participants with prior receipt of dialysis or organ transplant, previously diagnosed with severe mood and psychotic diseases, missing data that was essential for MetS diagnostic criteria and cognitive function were excluded.

### Measurements and assessment of covariates

Standard questionnaires at the baseline visit evaluated demographic characteristics, including age, sex, educational status (low: < 12th grade; high: ≥ 12th grade), smoking status (yes or no), alcohol status (yes or no), living status (living alone or not), and medical, medication history. Diabetes mellitus was diagnosed as HbA1c ≥ 6.5%, random blood glucose ≥ 11.1 mmol/L, fasting blood glucose ≥ 7.0 mmol/L, or self-reported, or a medical record of responding diagnosis or medication (yes or no). Hypertension was diagnosed as systolic blood pressure ≥ 140 mmHg and/or diastolic blood pressure ≥ 90 mmHg, or self-reported, or a medical record of responding diagnosis or medication (yes or no). The following information of HD therapy was also recorded: HD vintages, dialysis frequencies (twice/thrice per week), dialysis modality, including hemodialysis, hemodialysis combined with hemofiltration (HD + HF), hemodialysis combined with hemoperfusion (HP, a modality for blood purification by binding molecules to adsorbent materials with HP cartridges, HD + HP), hemodialysis combined with HDF, HP (HD + HF + HP). Anthropometric measurements, including standing height, weight, waist circumference (WC), hip circumference (HC), systolic blood pressure (SBP), and diastolic blood pressure (DBP) were performed by two trained nephrologists before the initiation of hemodialysis.

### Biochemical measurements

All participants provided venous blood samples and were collected before the initiation of hemodialysis therapy, after fasting for 8–10 h. Fasting blood glucose, total cholesterol, triglyceride (TG), high-density lipoprotein (HDL) cholesterol, low-density lipoprotein (LDL) cholesterol, and other biochemical indicators were measured using the biochemistry analyzer.

### Assessment of cognitive impairment

Cognitive function was assessed using the Mini Mental State Examination (MMSE) questionnaire by professional doctors at one hour of dialysis treatment, in order to eliminate the influence of hemodialysis [24]. The MMSE contains five subscales: orientation to time and space, 0–10 points; registration, 0–3 points; attention and calculation, 0–3 points; recall, 0–3 points; and language, 0–11 points. The total MMSE score is calculated as the sum of the subscales, and ranges from 0 to 30 points. with lower scores denoting worse cognitive function. A score of 30–27 points means no cognitive dysfunction, a score < 27 on the MMSE can be diagnosed as mild cognitive impairment (MCI) [25].

### Assessment of MetS

MetS was identified by the Chinese Guidelines for the Prevention and Treatment. of Type 2 Diabetes (2020 Edition) [26], the revised National Cholesterol Education Program Adult Treatment Group (ATPIII) [27]. The diagnostic criteria in China (2020 Edition), wherein three or more can be considered MetS, are as follows: (1) abdominal obesity (central obesity): WC ≥ 90 cm for men and ≥ 85 cm for women; (2) hyperglycemia: fasting blood glucose ≥ 6.1 mmol/L or 2 h blood glucose after sugar load ≥ 7.8 mmol/L and those who have been diagnosed with diabetes and treated; (3) hypertension: blood pressure ≥ 130/85 mmHg (1 mmHg = 0.133 kPa) and (or) confirmed hypertension and treated; and (4) fasting triglycerides (TG) ≥ 1.70 mmol/L, (5) fasting HDL-C < 1.04 mmol/L. According to the definition of NCEP-ATPIII, MetS requires at least 3 of 5 components: (i) central obesity (WC: ≥ 90 cm in men and ≥ 80 cm in women); (ii) elevated TG (TG ≥ 1.7 mmol/L); (iii) low HDL-C (HDL-C: < 1.03 mmol/L in men, < 1.29 mmol/L in women); (iv) elevated blood pressure (systolic/diastolic ≥ 130/85 mmHg, or use antihypertensive drugs); and (v) hyperglycemia (FPG ≥ 5.6 mmol/L or previously diagnosed with diabetes).

### Calculation of metabolic-related indices

siMS score was calculated as [28]:siMS score = 2 × waist/height + fasting glucose/5.6 + TG/1.7 + SBP/130− HDL/1.02 (male) or 1.28 (female).

CMI was calculated as [29]:

CMI = TG/HDL-C × WHtR.

MetSSS was calculated as [23]:

MetSSS =  − 8.2939 + 0.0126 × FPG + 0.0063 × SBP + 0.0382 × WC− 0.0210 × HDL-c 0.8432 × ln(TG) (if male).

MetSSS =  − 7.5210 + 0.0156 × FPG + 0.0073 × SBP + 0.0292 × WC− 0.0207 × HDL-c 0.9065 × ln(TG) (if female).

### Statistical analysis

Characteristics of participants were presented as mean (standard deviation, SD) and median (interquartile range, IQR), respectively, for continuous variables that were normally and non-normally distributed by Kolmogorov–Smirnov test and numbers and proportions for categorical variables. Comparisons between groups were performed using Student’s *t*-test, chi-square test or Mann–Whitney U test when appropriate.

Univariate and multivariate binary logistic regression analyses were conducted to determine the relationship between MetS (2020 Edition), its components and related indices and the prevalence of MCI. Univariate and multivariate linear regression analyses were performed to explore the associations with MMSE score and five cognitive domains. Data were summarized as odds ratios (ORs), unstandardized coefficient (β), and regression coefficients (95%CIs), respectively. The adjustments were made for patients age, sex, educational level, smoking history, alcohol history, living alone, dialysis vintage, dialysis modality, dialysis access, and hemoglobin levels. In order to further examine the independent association between each MetS component and MCI, a mutually adjusted model was created by including other 4 MetS components as continuous variables as well as the covariates in Model 3, respectively. We also carried out restricted cubic spline analysis to detect the nonlinear dose–response relationship between three metabolic scores and the risk of MCI, with three knots placing at the 10th, 50th, and 90th percentiles. We further conducted sensitivity analyses by diagnosing MetS with ATPIII criteria on the full multivariable logistic and linear regression models. Subgroup analysis was conducted by age (< 65 or ≥ 65 years), sex (male or female), educational level (low or high), smoking history (yes or no), alcohol history (yes or no), living alone (yes or no), and dialysis modality (HD, HD + HDF, HD + HP, or HD + HDF + HP), and the potential interactions were evaluated.

All statistical analyses were performed by IBM SPSS Statistics (version 22.0, Chicago, USA) the statistical packages R (The R Foundation; http://ww.r-project.org; version 4.0.1). A two-sided *P* value < 0.05 was considered statistically different.

## Results

### Characteristics of study participants

**Fig. 1:**
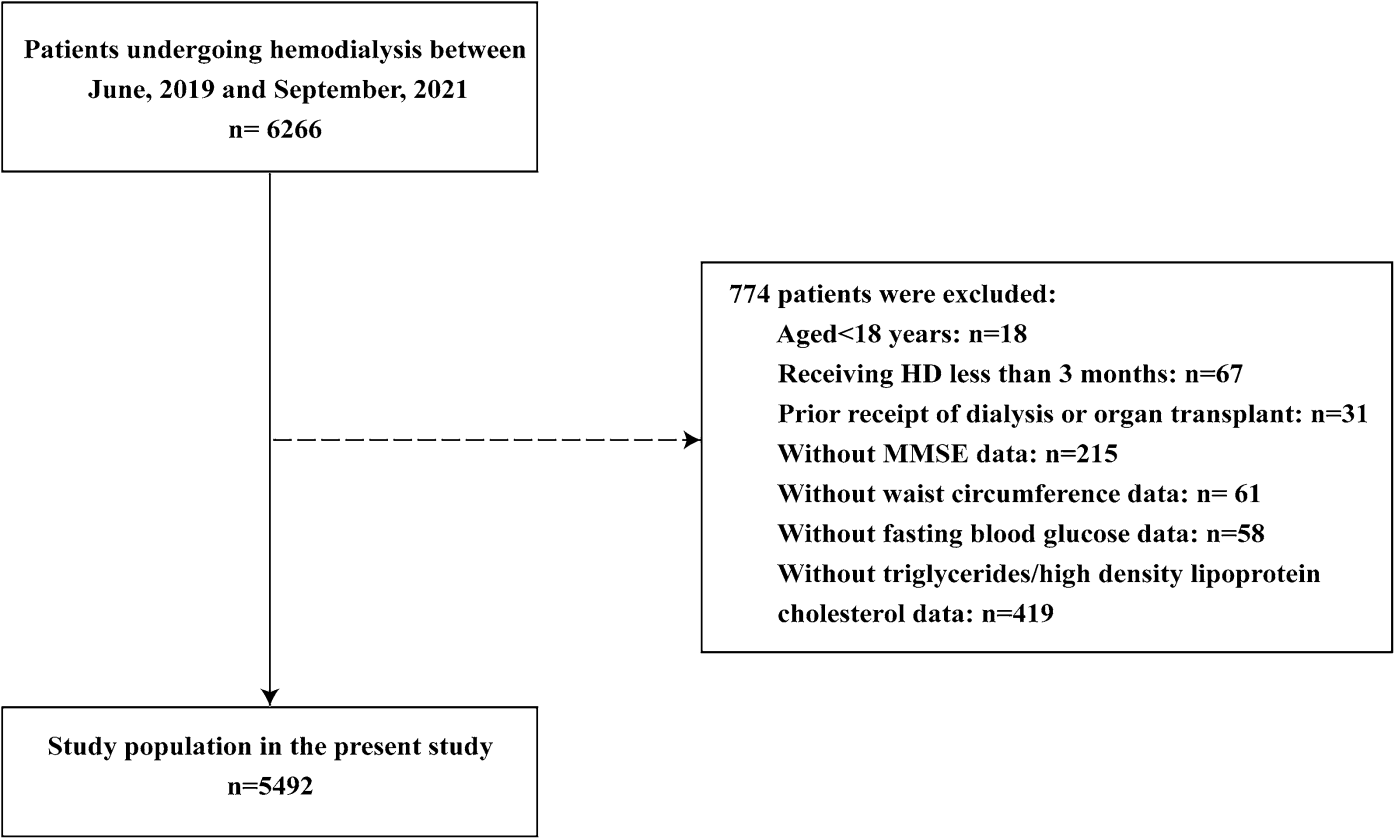
Flowchart of this study. HD, hemodialysis

As demonstrated in the flow chart (Fig. 1), a total of 5492 hemodialysis patients with available information on MetS and cognitive function were included in the final analyses. Of the included participants, the mean age was 54.4 ± 15.2 years old, 3351 (61.0%) were men. 71.6% (3933) patients undergo hemodialysis in a combined modality of HD, HDF, and HP, 86.2% (4733) dialyzed with fistula as the access, with the median dialysis vintage was 37.0(15.0,70.0) months. The mean number of MetS component was 2.8 ± 1.1, and the prevalence of MetS (three components and more) was 57.6%. The mean MMSE score was 26.9 ± 3.8, and the prevalence of MCI (MMSE < 27) was 34.3%. The patients were stratified into two groups according to Normal cognition group (n = 3609, 65.7%) or MCI group (n = 1883, 34.3%).

A comparison of the clinical characteristics between two groups is shown in Table 1. Compared to the patients with normal cognition, those with MCI were older, more likely female, with alcohol history, lower educational level, less likely living alone, higher prevalence rates of diabetes mellitus and hypertension, lower DBP levels, higher waist, hip circumference, higher fasting glucose levels, and lower hemoglobin, creatinine levels. Regarding MetS related parameters, the patients with MCI had higher prevalence of MetS, abdominal obesity, hyperglycemia, and higher levels of siMS score, CMI and MetSSS (all *P* < 0.05).

**Table 1:** Baseline characteristics of hemodialysis patients according to cognitive function

Characteristics	All (n = 5492)	Normal cognition (n = 3609)	MCI (n = 1883)	*P*
MMSE score	26.9 ± 3.8	29.2 ± 1.1	22.7 ± 2.6	< 0.001
Age (year)	54.4 ± 15.2	52.4 ± 15.0	58.2 ± 14.9	< 0.001
Female sex (n, %)	2141(39.0%)	1309(26.3%)	832(44.2%)	< 0.001
Smoking history (n, %)	1533 (27.9%)	1021 (28.3%)	512 (27.2%)	0.392
Alcohol history (n, %)	485 (8.8%)	291 (8.1%)	194 (10.3%)	0.006
High educational level (n, %)	2205 (40.1%)	1527 (42.3%)	678 (36.0%)	< 0.001
Living alone (n, %)	4152 (75.6%)	2876 (79.7%)	1276 (67.8%)	< 0.001
Diabetes mellitus (n, %)	1512 (27.5%)	906 (25.1%)	606 (32.3%)	< 0.001
Hypertension (n, %)	4336 (79.0%)	2777 (76.9%)	1559 (82.8%)	< 0.001
Dialysis vintage (months)	37.0 (15.0, 70.0)	37.0 (15.0, 72.0)	37.0 (15.0, 67.0)	0.356
Dialysis modality (n, %)				0.334
HD	490 (8.9%)	312 (8.6%)	178 (9.5%)	
HD + HDF	678 (12.3%)	462 (12.8%)	216 (11.5%)	
HD + HP	391 (7.1%)	249 (6.9%)	142 (7.5%)	
HD + HDF + HP	3933 (71.6%)	2586 (71.7%)	1347 (71.5%)	
Dialysis access, Fistula (n, %)	4733 (86.2%)	3139 (87.0%)	1594 (84.7%)	0.108
SBP (mmHg)	138.1 ± 20.6	138.1 ± 20.2	138.2 ± 21.2	0.844
DBP (mmHg)	78.6 ± 13.6	97.6 ± 13.6	76.7 ± 13.5	< 0.001
Body height (cm)	161.0 ± 8.2	161.8 ± 8.1	159.5 ± 8.3	< 0.001
Body weight (kg)	47.4 ± 19.8	49.9 ± 19.2	42.8 ± 20.0	< 0.001
Waist circumference (cm)	83.3 ± 10.8	82.8 ± 10.7	84.3 ± 10.8	< 0.001
Hip circumference (cm)	89.5 ± 8.1	89.3 ± 8.1	89.8 ± 8.1	0.025
Fasting glucose (mmol/L)	7.5 ± 3.8	7.4 ± 3.7	7.7 ± 4.0	0.012
Hemoglobin (g/L)	108.4 ± 20.6	109.0 ± 20.4	107.5 ± 21.0	0.011
TG (mmol/L)	1.51 (1.06, 2.31)	1.49 (1.05, 2.29)	1.55 (1.07, 2.36)	0.060
Cholesterol (mmol/L)	3.90 ± 0.96	3.88 ± 0.97	3.93 ± 0.96	0.059
HDL-c (mmol/L)	1.11 (0.91, 1.35)	1.11 (0.91, 1.35)	1.09 (0.90, 1.35)	0.556
LDL-c (mmol/L)	2.08 (1.63, 2.60)	2.07 (1.63, 2.59)	2.10 (1.63, 2.63)	0.236
MetS (2020 Edition) (%)	3161 (57.6%)	1996 (55.3%)	1165 (61.9%)	< 0.001
MetS component	2.8 ± 1.1	2.7 ± 1.1	2.9 ± 1.1	< 0.001
Abdominal obesity (n, %)	1818 (33.1%)	1112 (30.8%)	706 (37.5%)	< 0.001
Hypertriglyceridemia (n, %)	2384 (43.4%)	1533 (42.5%)	851 (45.2%)	0.055
Low HDL-c (n, %)	2346 (42.7%)	1524 (42.2%)	822 (43.7%)	0.315
Hyperglycemia (n, %)	3550 (64.6%)	2259 (62.6%)	1291 (68.6%)	< 0.001
High blood pressure (n, %)	5117 (94.3%)	3388 (93.9%)	1789 (95.0%)	0.087
siMS score	3.52 ± 1.28	3.47 ± 1.24	2.62 ± 1.37	< 0.001
CMI	0.70 (0.44, 1.21)	0.68 (0.43, 1.18)	0.75 (0.44, 1.26)	0.004
MetSSS	0.83 (0.08, 1.70)	0.75 (0.05, 1.62)	0.98 (0.19, 1.88)	< 0.001

*P* < 0.05 was considered statistically significant. Values were expressed as mean ± SD, median (25th–75th percentile), or frequency (percentage) as appropriate. *MCI* Mild cognitive impairment, *MMSE* Mini-mental state examination, *HD* Hemodialysis, *HDF* Hemofiltration; HP Hemoperfusion, SBP Systolic blood pressure, *DBP* Diastolic blood pressure, *TG* Triglyceride, *HDL* High density lipoprotein, LDL Low density lipoprotein, *MetS* Metabolic syndrome, *siMS* Metabolic syndrome score, *CMI* Cardiometabolic index, *MetSSS* Metabolic syndrome severity score

### Associations of MetS and severity scores with MMSE, and specific cognitive function domains

The associations between MetS (2020 Edition) and MMSE score in linear regression analyses are shown Table 2. After adjusting age, sex, educational level, smoking history, alcohol history, living alone, dialysis vintage, dialysis modality, dialysis access, and hemoglobin levels, MetS were significantly associated with lower MMSE score (*P* < 0.001). Regarding three metabolic severity scores, siMS score (*P* = 0.002), CMI (*P* = 0.001) and MetSSS (*P* < 0.004) were all associated with lower MMSE score.

**Table 2:** Association of MetS (2020 Edition) and metabolic scores with MMSE score, and specific cognitive domains using linear regression analysis among hemodialysis patients

Characteristics	Model 1		Model 2		Model 3	
	β (95%CI)	*P*	β (95%CI)	*P*	β (95%CI)	*P*
MetS (2020 Edition)						
MMSE score	− 0.496 (− 0.700, − 0.292)	< 0.001	− 0.317 (− 0.517, − 0.117)	0.002	− 0.324 (− 0.523, − 0.125)	< 0.001
Orientation	− 0.108 (− 0.172, − 0.044)	0.001	− 0.074 (− 0.138, − 0.011)	0.022	− 0.075 (− 0.139, − 0.012)	0.020
Registration	− 0.039 (− 0.063, − 0.014)	0.002	− 0.026 (− 0.051, − 0.001)	0.040	− 0.026 (− 0.051, − 0.002)	0.036
Attention and calculation	− 0.068 (− 0.150,0.014)	0.102	− 0.015 (− 0.096, 0.066)	0.712	− 0.025 (− 0.105, 0.056)	0.550
Recall	0.091 (− 0.139, − 0.042)	< 0.001	− 0.056 (− 0.104, − 0.007)	0.024	− 0.056 (− 0.104, − 0.008)	0.021
Language	− 0.188 (− 0.257, − 0.119)	< 0.001	− 0.143 (− 0.211, − 0.074)	< 0.001	− 0.141 (− 0.209, − 0.072)	< 0.001
siMS score						
MMSE score	− 0.192 (− 0.270, − 0.113)	< 0.001	− 0.126 (− 0.203, − 0.049)	0.001	− 0.123 (− 0.199,− 0.046)	0.002
Orientation	− 0.055 (− 0.080, − 0.030)	< 0.001	− 0.042 (− 0.066, − 0.017)	0.001	− 0.041 (− 0.065,− 0.016)	0.001
Registration	− 0.013 (− 0.023, − 0.004)	0.007	− 0.009 (− 0.018, 0.001)	0.073	− 0.009 (− 0.018, 0.001)	0.078
Attention and calculation	− 0.036 (− 0.068, − 0.005)	0.025	− 0.011 (− 0.042, 0.020)	0.476	− 0.012 (− 0.043, 0.019)	0.434
Recall	− 0.021 (− 0.040, − 0.003)	0.025	− 0.012 (− 0.031, 0.006)	0.194	− 0.011 (− 0.030, 0.007)	0.227
Language	− 0.059 (− 0.086, − 0.033)	< 0.001	− 0.046 (− 0.072, − 0.019)	0.001	− 0.044 (− 0.071, − 0.018)	0.001
CMI						
MMSE score	− 0.213 (− 0.312, − 0.114)	< 0.001	− 0.166 (− 0.263, − 0.070)	0.001	− 0.160 (− 0.256, − 0.065)	0.001
Orientation	− 0.076 (− 0.107, − 0.045)	< 0.001	− 0.066 (− 0.097, − 0.036)	< 0.001	− 0.066 (− 0.096, − 0.036)	< 0.001
Registration	− 0.012 (− 0.024, 0.000)	0.050	− 0.009 (− 0.073, − 0.023)	< 0.001	− 0.008 (− 0.020, 0.004)	0.168
Attention and calculation	− 0.024 (− 0.064, 0.015)	0.229	− 0.008 (− 0.047, 0.031)	0.685	− 0.006 (− 0.045, 0.032)	0.750
Recall	− 0.016 (− 0.039, 0.008)	0.184	− 0.009 (− 0.032, 0.015)	0.469	− 0.007 (− 0.030, 0.017)	0.579
Language	− 0.080 (− 0.114, − 0.047)	< 0.001	− 0.070 (− 0.103, − 0.037)	< 0.001	− 0.069 (− 0.102, − 0.036)	< 0.001
MetSSS						
MMSE score	− 0.231 (− 0.303, − 0.159)	< 0.001	− 0.106 (− 0.180,-0.033)	0.005	− 0.107 (− 0.180, − 0.034)	0.004
Orientation	− 0.054 (− 0.076, − 0.031)	< 0.001	− 0.027 (− 0.050, − 0.004)	0.022	− 0.027 (− 0.050, − 0.004)	0.023
Registration	− 0.018 (− 0.026, − 0.009)	< 0.001	− 0.009 (− 0.018, 0.000)	0.043	− 0.009 (− 0.019, − 0.000)	0.042
Attention and calculation	− 0.064 (− 0.093, − 0.035)	< 0.001	− 0.011 (− 0.040, 0.019)	0.474	− 0.014 (− 0.043, 0.016)	0.355
Recall	− 0.028 (− 0.045, − 0.011)	0.001	− 0.016 (− 0.033, 0.001)	0.067	− 0.013 (− 0.031, 0.004)	0.132
Language	− 0.064 (− 0.087, − 0.039)	< 0.001	− 0.040 (− 0.065, − 0.015)	0.002	− 0.039 (− 0.064, − 0.014)	0.002

Model 1, crude model; Model 2, adjusted for age, sex; Model 3, adjusted for age, sex, educational level, smoking history, alcohol history, living alone, dialysis vintage, dialysis modality, dialysis access, and hemoglobin levels. *MetS* Metabolic syndrome, *MMSE* Mini-mental state examination, *β* Unstandardized coefficient, *CI* Confidence interval, *siMS* Metabolic syndrome score,* CMI* Cardiometabolic index, *MetSSS* Metabolic syndrome severity score

The relationships between MetS (2020 Edition) and five specific cognitive function domains were also evaluated in this study (Table 2). The results found that orientation (*P* = 0.020), registration (*P* = 0.036), recall (*P* = 0.021), and language (*P* < 0.001) domains were inversely related with the prevalence of MetS, but there was no association between attention and calculation domain and MetS (*P* = 0.550), after adjusting clinical covariates. Orientation, and language domains were also associated with siMS score, CMI and MetSSS (all *P* < 0.05); Attention and calculation domain, and recall domain were not associated with three severity scores, and only registration was positively related with MetSSS (*P* = 0.042).

### Association of MetS, its components, numbers and severity scores with the prevalence of MCI

This study further explored the association between metabolism and the prevalence of MCI (Table 3). The results demonstrated that MetS (2020 Edition), abdominal obesity, and hyperglycemia had higher risk of MCI with multivariate-adjusted ORs of 1.22 (95% CI 1.08–1.37, *P* = 0.001), 1.14(1.01–1.28, *P* = 0.039), and 1.17(1.04–1.33, *P* = 0.011), respectively. However, hypertriglyceridemia (*P* = 0.089), low HDL-cholesterol (*P* = 0.079) and high blood pressure (*P* = 0.093) were not associated with MCI. Further, after adjusting other MetS components, only the significant association between abdominal obesity and MCI was still observed (OR, 1.15; 95% CI 1.01–1.30; *P* = 0.031). Compared with those without MetS components, patients with 2, 3, 4, 5 MetS components had 2.03-fold (95% CI 1.04–3.98), 2.51-fold (1.28–4.90), 2.35-fold (1.20–4.62), and 2.94-fold (1.48–5.84) risk of MCI, respectively (all *P* < 0.05).

**Table 3:** Association of MetS (2020 Edition), its components, number and metabolic scores with incident MCI using logistic regression analysis among hemodialysis patients

Characteristics	Model 1		Model 2		Model 3		Mutual model	
	OR(95%CI)	*P*	OR(95%CI)	*P*	OR(95%CI)	*P*	OR(95%CI)	*P*
MetS (2020 Edition)	1.31 (1.17, 1.47)	< 0.001	1.21 (1.08, 1.36)	0.001	1.22 (1.08, 1.37)	0.001	–	
MetS component								
Abdominal obesity (%)	1.35 (1.20, 1.51)	< 0.001	1.14 (1.01, 1.29)	0.032	1.14 (1.01, 1.28)	0.039	1.15 (1.01, 1.30)	0.031
Hypertriglyceridemia (%)	1.12 (0.99, 1.25)	0.054	1.10 (0.98, 1.23)	0.105	1.11 (0.99, 1.24)	0.089	1.09 (0.97, 1.23)	0.145
Low HDL-c (%)	1.06 (0.95, 1.19)	0.311	1.11 (0.99, 1.24)	0.088	1.11 (0.99, 1.25)	0.079	1.04 (0.85, 1.27)	0.695
Hyperglycemia (%)	1.30 (1.16, 1.47)	< 0.001	1.18 (1.04, 1.33)	0.010	1.17 (1.04, 1.33)	0.011	1.28 (0.98, 1.68)	0.070
High blood pressure (%)	1.24 (0.97, 1.59)	0.087	1.24 (0.96, 1.59)	0.099	1.24 (0.96, 1.60)	0.093	1.09 (0.97, 1.22)	0.168
Number of MetS components							–	
0	References		References		References			
1	1.73 (0.88, 3.40)	0.115	1.79 (0.90, 3.55)	0.098	1.86 (0.93, 3.71)	0.078		
2	1.99 (1.03, 3.87)	0.041	1.93 (0.99, 3.76)	0.055	2.03 (1.04, 3.98)	0.039		
3	2.56 (1.32, 4.95)	0.005	2.36 (1.21, 4.60)	0.012	2.51 (1.28, 4.90)	0.007		
4	2.60 (1.34, 5.05)	0.005	2.24 (1.15, 4.38)	0.018	2.35 (1.20, 4.62)	0.013		
5	3.52 (1.79, 6.91)	< 0.001	2.78 (1.40, 5.51)	0.003	2.94 (1.48, 5.84)	0.002		
siMS score	1.09 (1.05, 1.14)	< 0.001	1.06 (1.02, 1.11)	0.007	1.06 (1.02, 1.11)	0.008		
CMI	1.10 (1.04, 1.16)	0.001	1.08 (1.02, 1.14)	0.006	1.08 (1.02, 1.14)	0.009		
MetSSS	1.12 (1.08, 1.16)	< 0.001	1.06 (1.02, 1.11)	0.007	1.06 (1.02, 1.11)	0.006		

Model 1, crude model; Model 2, adjusted for age, gender; Model 3, adjusted for age, gender, educational level, smoking history, alcohol history, living alone, dialysis vintage, dialysis modality, dialysis access, and hemoglobin levels. Mutual Model, adjusted for other MetS components as continuous variables based on Model 3, respectively. *MetS* Metabolic syndrome, *MCI* Mild cognitive impairment, *OR* Odds ratio, *CI* Confidence interval, *HDL* High density lipoprotein, *siMS* Metabolic syndrome score, *CMI* Cardiometabolic index, *MetSSS* Metabolic syndrome severity score

**Fig. 2:**
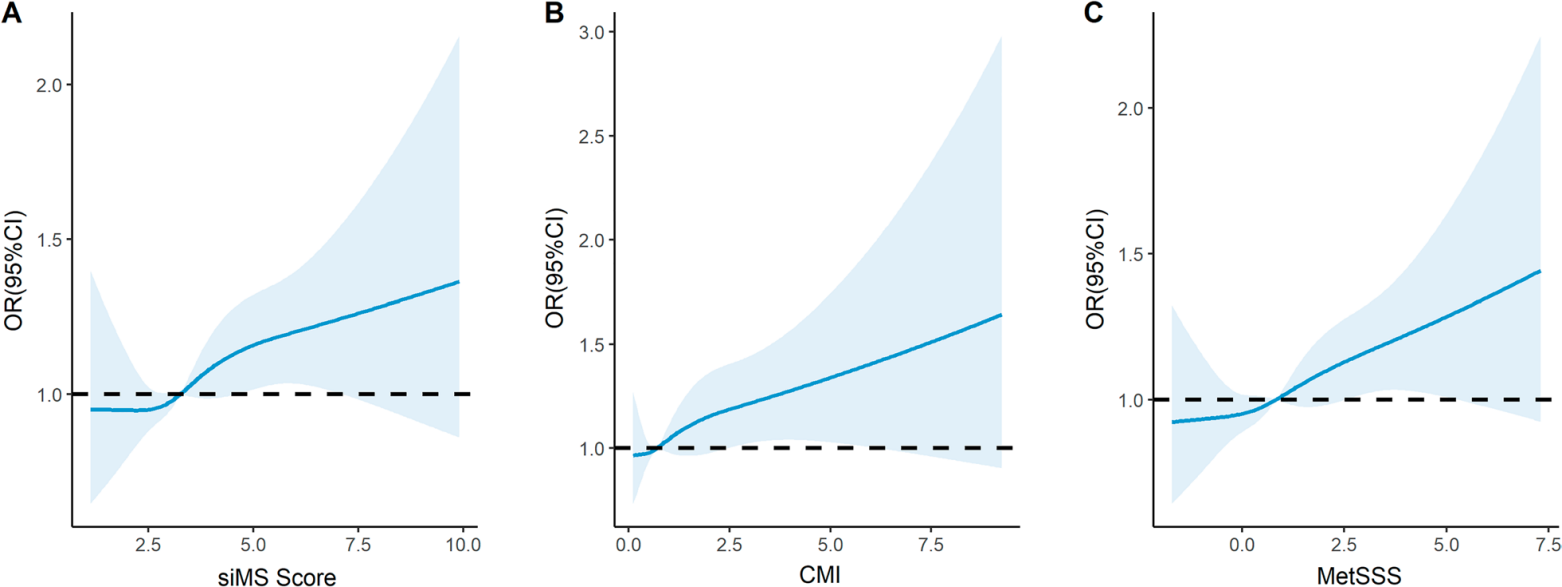
The dose–response relationship between siMS score, CMI, MetSSS and the risk of MCI for hemodialysis patients. Point estimates (solid line) and 95% confidence intervals (dashed lines) were estimated by restricted cubic splines analysis with knots placed at the 10th, 50th, and 90th percentile. Model was adjusted for age, sex, educational level, dialysis vintage, dialysis access, dialysis modality, smoking, alcohol history, and hemoglobin levels. *siMS* Metabolic syndrome score, *CMI* Cardiometabolic index, *MetSSS* Metabolic syndrome severity score

We also found that siMS score, CMI, and MetSSS were associated with higher risk of MCI with multivariate-adjusted ORs of 1.06 (95% CI 1.02–1.11, *P* = 0.008), 1.08(1.02–1.14, *P* = 0.009), and 1.06(1.02–1.11, *P* = 0.006), respectively. In the cubic spline models, no departure from linearity was found for the relationship between three metabolic scores and the risk of MCI (*P* for nonlinearity = 0.731 for siMS score; *P* for nonlinearity = 0.750 for CMI; and *P* for nonlinearity = 0.904 for MetSSS; Fig. 2).

### Subgroup analyses of association between MetS and incident MCI

**Fig. 3:**
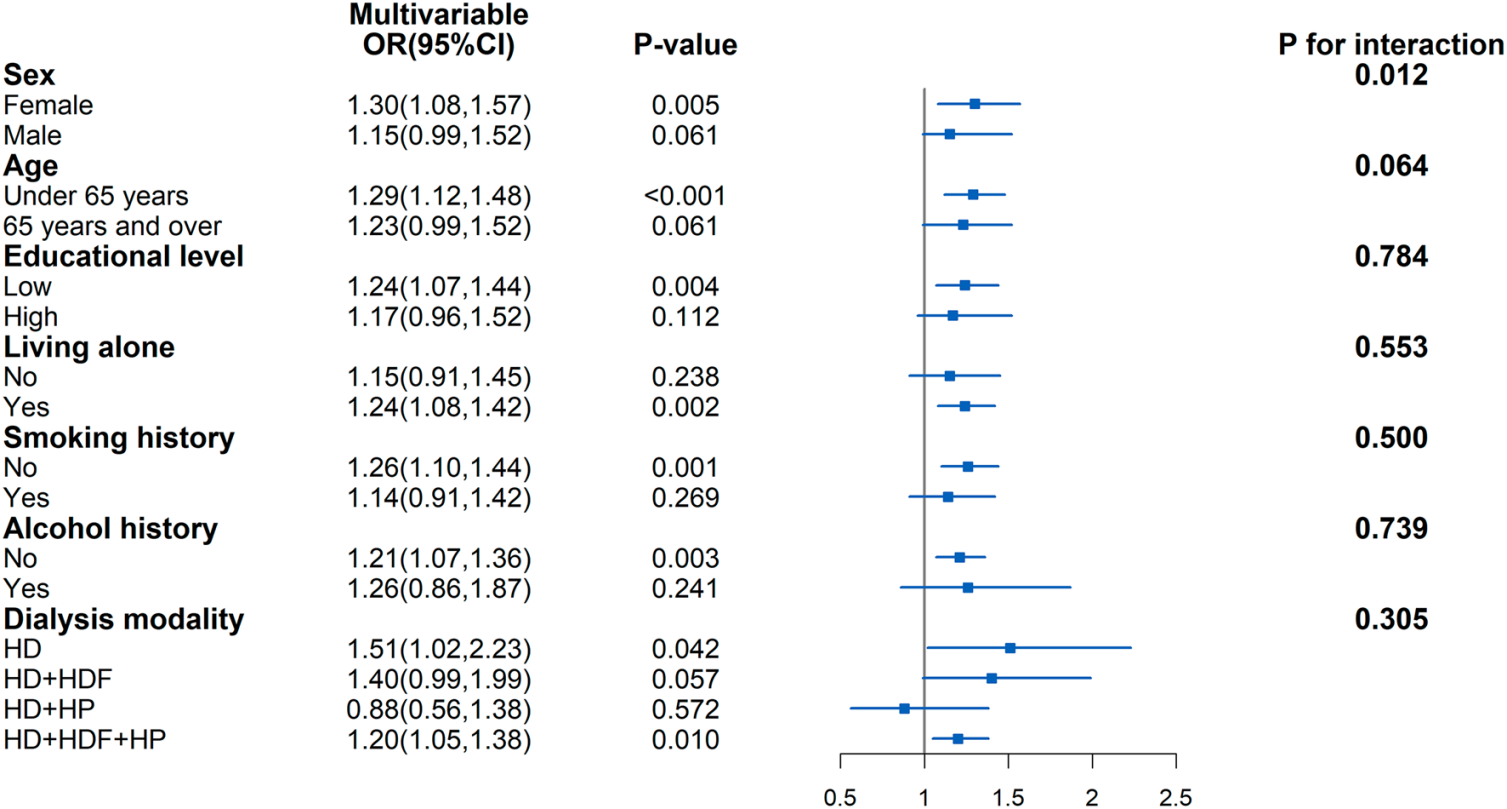
Subgroup analyses of association between MetS and MCI. Model was adjusted for age, sex, educational level, dialysis vintage, dialysis access, dialysis modality, smoking, alcohol history, and hemoglobin levels. *OR* Odds ratio, *CI* Confidence interval, *HD* Hemodialysis, *HDF* Hemofiltration, *HP* Hemoperfusion

A stronger association between MetS (2020 Edition) and incident MCI was found among female patients (OR 1.30, 95% CI 1.08–1.57, *P* = 0.012), aged < 65 years (OR 1.29, 1.12–1.48, *P* < 0.001), low educational level (OR 1.24, 1.07–1.44, *P* = 0.004), living alone (OR 1.24, 1.08–1.42, *P* = 0.002), non-smoking (OR 1.26, 1.10–1.44, *P* = 0.001), and HD as dialysis modality (OR 1.51, 1.02–2.23, *P* = 0.042). Significant interaction effect of sex on the MetS-MCI was observed (*P* for interaction = 0.012). Detailed information is shown in Fig. 3 and Additional file 1: Table S1.

### Sensitivity analysis

To further evaluate our findings, this study tested whether MetS, diagnosed with ATPIII criteria, was also associated with MCI. First, baseline characteristics of MetS (ATPIII) is shown in Additional file 1: Table S2. As expected, MetS (ATPIII) had similar relationships with MMSE score and the cognitive domains in the full adjusted linear regression model (all *P* < 0.05, Additional file 1: Table S3). Results were also comparable to the main analyses in logistic regression models, expect that there was independently significant association between high blood pressure and MCI (OR 1.30, 95% CI 1.10–1.53, *P* = 0.002) (Additional file 1: Table S4). Finally, the subgroup analyses were also performed and found that not only sex (*P* for interaction = 0.023), but also smoking history (*P* for interaction = 0.040) has significant interaction effects on the association between MetS (ATPIII) and MCI risk (Additional file 1: Table S5).

## Discussion

The main findings of this current study indicated that MetS, abdominal obesity, and hyperglycemia were associated with increased risk of MCI in hemodialysis patients. Metabolic severity scores, including metabolic syndrome score, cardiometabolic index, and metabolic syndrome severity score, were also significantly associated with increased risk of MCI. We found evidence of a dose-dependent association, with MCI risk increasing the number of components involved, up to 2.9-fold risk for 5 components. Further analysis showed that MetS was negatively associated with MMSE score, orientation, registration, recall and language. Each standard deviation increase of MetS was associated with a 32.4% lower MMSE score hazard. There was significant interaction effect of sex on the association between MetS and MCI. These associations between MetS and MCI, MMSE score were robust in the sensitivity analyses.

The first important finding of this study is that MetS and its components were risk factors for incident MCI risk in hemodialysis patients. Regarding the association with continuous MMSE score, it was approximately in accordance with above results. These results are in accordance with previous studies [16–20]. A cross-sectional study conducted in China reported that MetS was associated with cognitive impairment which was assessed by MMSE [30]. The French Three-City Study demonstrated that MetS was inversely associated with global cognitive decline assessed by a lower MMSE score [31]. A Taiwan large-scale study including 28486 elderly participants also explored the similar association between MetS (ATPIII) and cognitive impairment (MMSE < 24) [20].

The possible mechanisms underlying the association between MetS and impaired cognitive function could be as below. First, the brief exposure to MetS disturbances, such as hyperglycemic, and hyperlipidemic conditions, increases contractility of cerebral vascular smooth muscle cells [32], causes systemic and localized perivascular adipose tissue inflammation [33]. The changes lead to structural and functional changes of the brain, and cerebral hypoperfusion potentially precipitating cognitive dysfunction [34]. Second, MetS triggers a series of microvascular dysfunction, such as worse local insulin resistance, increased serum free fatty acids and low nitric oxide levels [35, 36]. These changes induce cerebrovascular vasoconstriction, cerebral blood flow reduction, and increase oxidative stress, which cause cerebral hypoperfusion, damage blood–brain barrier integrity, leading to cognitive dysfunction [37]. Third, one of common complications in MetS is the perturbance of neuronal homeostatic processes through the activation of various inflammatory signaling pathways, including autophagy, apoptosis, and neurogenesis, which leads to cognitive deterioration [36, 38, 39].

This study also found that abdominal obesity, and hyperglycemia were the main MetS components which were associated with MCI. This is consistent with previous findings supporting a greater impact of specific risk factor contributors in the association between MetS and cognitive impairment [30, 40–42]. Although distinct results were seen when the association between individual components of MetS and cognitive impairment were investigated, abdominal obesity, and hyperglycemia are acknowledged factors for cognitive impairment in the general population [43]. Hemodialysis population share most of these same risk factors for cognitive impairment. In addition, this current study found that central obesity was the only independent risk factor for the MCI when evaluating MetS with diagnostic criteria in China (2020 Edition), even adjusting for other MetS components. This finding takes our pinpointing of amenable factors for MCI a step further in more precise populations, which may be useful for early prevention and control of MCI progression among hemodialysis patients.

We examined the associations of novel metabolic related scores with cognitive function. The findings demonstrated that siMS score, CMI and metSSS are all associated with increased risk of MCI and lower MMSE score. These scores have been proven optimal methods for quantification of MetS, and the severity [21–23]. Previous studies reported that they were associated with increased risk of stroke, cardiovascular diseases, all-cause mortality [44–47]. To our best knowledge, no studies have focused on the associations with cognition. This was the first study to assess the predictive effects of these scores on cognitive impairment. It implies that continuous metabolic score may be a convenient option for predicting cognitive decline for hemodialysis in clinics.

The second important finding of this study is that MetS was negatively associated with not only global cognitive function but also almost all specific functions. MetS significantly predicted poor orientation, memory, and language performance, even after controlling for traditional risk factors. A prospective cohort study including 2880 middle-aged community-dwelling adults found that MetS is associated with worse cognition mainly in domains of verbal memory and verbal fluency [18], which verified our results. Przybyien et al. also found that MetS had a negative cross-sectional association of MetS with global cognition and memory [19]. The following clinically relevant links can explain our results: cognitive function is related to the orbitofrontal cortex, temporal lobe and hippocampus, which are essential of cognitive maintenance on the memory, language and orientation [48–50]. On the flip side, frontal cortex and hippocampus are potential target cerebral regions which are rich in insulin receptors [51]. Hyperglycemia, as the MetS component, may affect amyloid processing and increase brain intraneuronal b-amyloid deposition [52] and tau hyperphosphorylation [53] in target regions, which is a sign of cognitive impairment. In addition, a previous study found that individuals with abdominal obesity had a significantly lower density of gray matter in the frontal lobe, post-central gyrus, and middle frontal gyrus [54], which exacerbates cognitive impairment. These findings underscore the importance of metabolism in maintaining healthy cognitive function domains.

The third important finding of this study is that the number of MetS components increased with higher prevalence of MCI, with a positive dose–response effect. Similar results were found in a Chinese longitudinal study, which demonstrated that the count of metabolic factors was associated with worse declines in cognition [19]. Another study demonstrated that rather than the diagnose of the MetS itself, the count of the MetS risk factors was more useful in discriminating risk of cognitive impairment in aging adults [55]. This current study found that compared with those without MetS components, patients with only two components of MetS had 2.03-fold increased risk of MCI, even they did still meet MetS criteria, and the OR in the multivariate logistic regression models significantly increased according to the number of MetS components, until with 5 components, patients had 2.94-fold increased risk of MCI. These results suggest that individual small effects of single MetS components are amplified when occurring together in the same patients as the MetS. Hence, early prevention and intervention of each component of MetS is very essential to reduce the risk of MCI prevalence, even if MetS has not yet been diagnosed. On the other hand, clinical physicians should also spare no effort to improve each abnormal component and decrease the total number of MetS components for patients who have been diagnosed as MetS.

Finally, the fourth important finding of this study is that sex have the interaction on the association between MetS and MCI. Cognitive function of women declined faster than those of men as growing old, sex-specific differences have been found in previous studies [56, 57], which is similar with our results. This finding emphasizes the effect of sex on MCI in the specific population.

The strengths of our study included the following: it is the first multicenter large-scale study to examine the relationship between MetS, metabolic scores and MCI, confirm the dose–response relationship among hemodialysis patients. Secondly, the study assessed the association between MetS and metabolic scores and specific cognitive function domains in hemodialysis patients. Thirdly, most recognized confounders were taken into account in regression models to analyze the independent association of MetS and MCI in this study. However, some limitations also exist. First, all participants in the present study come from a province of Southwestern China, which means that this study has a certain degree of regional limitation. Second, this study is based on a cross-sectional design, so it is not possible to determine causal relationships. To clarify this issue, a further longitudinal study is needed to be carried out to verify the relationship between MetS and MCI. Third, this study measured cognitive function only with the MMSE testing, and specific cognitive domains were not measured by corresponding test tools. Finally, despite a great quantity of potentially confounding factors having been adjusted, and the nature of all observational studies, some undetected and unmeasured confounders still cannot be excluded, such as the use of hypoglycemic drugs, lipid regulators and nutraceuticals, which may impact the risk of MetS components, and the family history of emotional, mental diseases or cognitive impairment, considering that they may have a certain genetic tendency.

## Conclusion

MetS and severity scores were associated with MCI, specific cognitive domains in hemodialysis patients, and there was a positive association between MetS components and MCI with a positive dose–response effect. Generally, our findings give us some inspiration on how to manage MetS and interfere with MCI in hemodialysis patients.

## Supplementary Information

**Additional file1: Table S1.** Subgroup analyses of the association between MetS (2020 Edition) and MCI among hemodialysis patients. **Table S2**. Baseline characteristics of MetS (ATPIII) in hemodialysis patients according to cognitive function. **Table S3.** Association of MetS (ATPIII) with MMSE score, and specific cognitive domains using linear regression analysis among hemodialysis patients. **Table S4.** Association of MetS (ATPIII), its components, number with incident MCI using logistic regression analysis among hemodialysis patients. **Table S5.** Subgroup analyses of the association between MetS (ATPIII) and MCI among hemodialysis patients.

## Data Availability

The datasets generated during the current study are available from the corresponding author upon reasonable request.
