# Supplementary material for: Associations of metabolic syndrome, its severity with cognitive impairment among hemodialysis patients
Source: Diabetol Metab Syndr. 2023 May 23;15:108. doi: 10.1186/s13098-023-01080-3 (PMC10204216; doi:10.1186/s13098-023-01080-3)
Supplement: Supplementary file 1 — Additional file1: Table S1. Subgroup analyses of the association between MetS (2020 Edition) and MCI among hemodialysis patients. Table S2. Baseline characteristics of MetS (ATPIII) in hemodialysis patients according to cognitive function. Table S3. Association of MetS (ATPIII) with MMSE score, and specific cognitive domains using linear regression analysis among hemodialysis patients. Table S4. Association of MetS (ATPIII), its components, number with incident MCI using logistic regression analysis among hemodialysis patients. Table S5. Subgroup analyses of the association between MetS (ATPIII) and MCI among hemodialysis patients. [file 13098_2023_1080_MOESM1_ESM.docx]

**Supplementary Materials**

**Table S1. Subgroup analyses of the association between MetS (2020 Edition) and MCI among hemodialysis patients.**

| **Characteristics** | **Crude Model** | | **Adjusted Model^*^** | | ***P* for interaction** |
| --- | --- | --- | --- | --- | --- |
|  | **OR(95%CI）** | ***P*** | **OR(95%CI）** | ***P*** |  |
| **Sex** |  |  |  |  | 0.012 |
| Female | 1.45(1.21,1.73) | <0.001 | 1.30(1.08,1.57) | 0.005 |  |
| Male | 1.23(1.06,1.43) | 0.006 | 1.15(0.99,1.34) | 0.069 |  |
| **Age** |  |  |  |  | 0.064 |
| <65 years | 1.29(1.12,1.48) | <0.001 | 1.29(1.12,1.48) | <0.001 |  |
| ≥65 years | 1.24(1.00,1.53) | 0.048 | 1.23(0.99,1.52) | 0.061 |  |
| **Educational level** |  |  |  |  | 0.784 |
| Low | 1.34(1.16,1.55) | <0.001 | 1.24(1.07,1.44) | 0.004 |  |
| High | 1.30(1.08,1.57) | 0.006 | 1.17(0.96,1.52) | 0.112 |  |
| **Living alone** |  |  |  |  | 0.553 |
| No | 1.20(0.96,1.50) | 0.108 | 1.15(0.91,1.45) | 0.238 |  |
| Yes | 1.30(1.14,1.49) | <0.001 | 1.24(1.08,1.42) | 0.002 |  |
| **Smoking history** |  |  |  |  | 0.500 |
| No | 1.34(1.18,1.54) | <0.001 | 1.26(1.10,1.44) | 0.001 |  |
| Yes | 1.23(0.99,1.53) | 0.061 | 1.14(0.91,1.42) | 0.269 |  |
| **Alcohol history** |  |  |  |  | 0.739 |
| No | 1.30(1.16,1.47) | <0.001 | 1.21(1.07,1.36) | 0.003 |  |
| Yes | 1.39(0.96,2.02) | 0.083 | 1.26(0.86,1.87) | 0.241 |  |
| **Dialysis modality** |  |  |  |  | 0.305 |
| HD | 1.50(1.03,2.20) | 0.036 | 1.51(1.02,2.23) | 0.042 |  |
| HD+HDF | 1.58(1.13,2.22) | 0.008 | 1.40(0.99,1.99) | 0.057 |  |
| HD+HP | 1.00(0.66,1.52) | 0.997 | 0.88(0.56,1.38) | 0.572 |  |
| HD+HDF+HP | 1.29(1.12,1.47) | <0.001 | 1.20(1.05,1.38) | 0.010 |  |

^*^ Adjusted for age, gender, educational level, smoking history, alcohol history, living alone, dialysis vintage, dialysis modality, dialysis access, and hemoglobin levels. Abbreviations: MetS, metabolic syndrome; MCI, mild cognitive impairment; OR, odds ratio; CI, confidence interval.

**Table S2**. **Baseline characteristics of MetS (ATPIII) in hemodialysis patients according to cognitive function**

| **Characteristics** | **All**  **(n=5492)** | **Normal cognition**  **(n=3609)** | **MCI**  **(n=1883)** | ***P*** |
| --- | --- | --- | --- | --- |
| MMSE score | 26.9±3.8 | 29.2±1.1 | 22.7±2.6 | <0.001 |
| MetS (ATPIII) (%) | 3419(62.3%) | 2144(59.4%) | 1275(67.7%) | <0.001 |
| MetS component | 2.9±1.2 | 2.8±1.2 | 3.1±1.2 | <0.001 |
| Abdominal obesity(n,%) | 4662(84.9%) | 1324(36.7%) | 847(45.0%) | <0.001 |
| Hypertriglyceridemia(n,%) | 2384(43.4%) | 1533(42.5%) | 851(45.2%) | 0.055 |
| Low HDL-c(n,%) | 2802(51.0%) | 1795(49.7%) | 1007(53.5%) | 0.009 |
| Hyperglycemia(n,%) | 3967(72.2%) | 2547(70.6%) | 1420(75.4%) | <0.001 |
| High blood pressure(n,%) | 4662(84.9%) | 3024(83.8%) | 1638(87.0%) | 0.002 |

Note: *P* < 0.05 was considered statistically significant. Values were expressed as mean±SD, median (25th–75th percentile), or frequency (percentage) as appropriate. Abbreviation: MetS, metabolic syndrome; MCI, mild cognitive impairment; MMSE, mini-mental state examination; HDL, high density lipoprotein.

**Table S3.** **Association of MetS (ATPIII) with MMSE score, and specific cognitive domains using linear regression analysis among hemodialysis patients.**

| **Characteristics** | **Model 1** | | **Model 2** | | **Model 3** | |
| --- | --- | --- | --- | --- | --- | --- |
|  | **β(95%CI）** | ***P*** | **β(95%CI）** | ***P*** | **β(95%CI）** | ***P*** |
| **MetS (ATPIII)** |  |  |  |  |  |  |
| MMSE score | -0.704(-0.912,-0.497) | <0.001 | -0.435(-0.641,-0.229) | <0.001 | -0.446(-0.651,-0.241) | <0.001 |
| Orientation | -0.158(-0.223,-0.093) | <0.001 | -0.102(-0.167,-0.037) | 0.002 | -0.105(-0.170,-0.040) | 0.002 |
| Registration | -0.051(-0.076,-0.026) | <0.001 | -0.033(-0.058,-0.007) | 0.012 | -0.033(-0.059,-0.008) | 0.010 |
| Attention and Calculation | -0.171(-0.255,-0.088) | <0.001 | -0.067(-0.150,0.016) | 0.115 | -0.079(-0.161,0.004) | 0.063 |
| Recall | -0.118(-0.168,-0.069) | <0.001 | -0.087(-0.136,-0.038) | 0.001 | -0.082(-0.131,-0.032) | 0.001 |
| Language | -0.206(-0.276,-0.136) | <0.001 | -0.151(-0.221,-0.080) | <0.001 | -0.150(-0.220,-0.079) | <0.001 |

Model 1, crude model; Model 2, adjusted for age, gender; Model 3, adjusted for age, gender, educational level, smoking history, alcohol history, living alone, dialysis vintage, dialysis modality, dialysis access, and hemoglobin levels. Abbreviation: MetS, metabolic syndrome; MMSE, mini-mental state examination; β, unstandardized coefficient; CI, confidence interval.

**Table S4. Association of MetS (ATPIII), its components, number with incident MCI using logistic regression analysis among hemodialysis patients.**

| **Characteristics** | **Model 1** | | **Model 2** | | **Model 3** | | **Mutual Model** | |
| --- | --- | --- | --- | --- | --- | --- | --- | --- |
|  | **OR(95%CI)** | ***P*** | **OR(95%CI)** | ***P*** | **OR(95%CI)** | ***P*** | **OR(95%CI)** | ***P*** |
| MetS (ATPIII) | 1.43(1.27,1.61) | <0.001 | 1.27(1.13,1.44) | <0.001 | 1.29(1.14,1.45) | <0.001 |  |  |
| MetS component |  |  |  |  |  |  |  |  |
| Abdominal obesity(%) | 1.41(1.26,1.58) | <0.001 | 1.13(1.01,1.28) | 0.042 | 1.13(1.00,1.28) | 0.044 | 1.10(0.98,1.25) | 0.112 |
| Hypertriglyceridemia(%) | 1.12(0.99,1.25) | 0.054 | 1.10(0.98,1.23) | 0.105 | 1.12(0.99,1.25) | 0.062 | 1.10(0.97,1.24) | 0.134 |
| Low HDL-c(%) | 1.16(1.04,1.30) | 0.008 | 1.12(0.99,1.26) | 0.057 | 1.12(0.99,1.26) | 0.067 | 1.09(0.97,1.23) | 0.145 |
| Hyperglycemia(%) | 1.28(1.12,1.45) | <0.001 | 1.17(1.03,1.33) | 0.019 | 1.17(1.02,1.33) | 0.022 | 1.14(0.99,1.30) | 0.060 |
| High blood pressure(%) | 1.29(1.10,1.52) | 0.002 | 1.28(1.08,1.51) | 0.004 | 1.29(1.10,1.53) | 0.002 | 1.30(1.10,1.53) | 0.002 |

Model 1, crude model; Model 2, adjusted for age, gender; Model 3, adjusted for age, gender, educational level, smoking history, alcohol history, living alone, dialysis vintage, dialysis modality, dialysis access, and hemoglobin levels. Mutual Model, adjusted for other MetS components as continuous variables based on Model 3, respectively. Abbreviation: MetS, metabolic syndrome; MCI, mild cognitive impairment; OR, odds ratio; CI, confidence interval; HDL, high density lipoprotein.

**Table S5. Subgroup analyses of the association between MetS (ATPIII) and MCI among hemodialysis patients.**

| **Characteristics** | **Crude Model** | | **Adjusted Model^*^** | | ***P* for interaction** |
| --- | --- | --- | --- | --- | --- |
|  | **OR(95%CI）** | ***P*** | **OR(95%CI）** | ***P*** |  |
| **Sex** |  |  |  |  | 0.023 |
| Female | 1.65(1.36,2.02) | <0.001 | 1.50(1.22,1.84) | <0.001 |  |
| Male | 1.24(1.07,1.44) | 0.005 | 1.18(1.02,1.38) | 0.030 |  |
| **Age** |  |  |  |  | 0.552 |
| <65 years | 1.42(1.24,1.64) | <0.001 | 1.38(1.20,1.60) | <0.001 |  |
| ≥65 years | 1.32(1.06,1.64) | 0.013 | 1.22(0.97,1.52) | 0.089 |  |
| **Educational level** |  |  |  |  | 0.384 |
| Low | 1.50(1.30,1.75) | <0.001 | 1.31(1.12,1.53) | 0.001 |  |
| High | 1.35(1.11,1.64) | 0.002 | 1.24(1.02,1.51) | 0.031 |  |
| **Living alone** |  |  |  |  | 0.218 |
| No | 1.57(1.24,1.99) | <0.001 | 1.44(1.13,1.84) | 0.003 |  |
| Yes | 1.33(1.16,1.52) | <0.001 | 1.24(1.08,1.43) | 0.003 |  |
| **Smoking history** |  |  |  |  | 0.040 |
| No | 1.55(1.35,1.78) | <0.001 | 1.38(1.20,1.60) | <0.001 |  |
| Yes | 1.18(0.95,1.48) | 0.131 | 1.08(0.86,1.35) | 0.504 |  |
| **Alcohol history** |  |  |  |  | 0.674 |
| No | 1.42(1.26,1.61) | <0.001 | 1.27(1.12,1.45) | <0.001 |  |
| Yes | 1.55(1.06,2.27) | 0.023 | 1.37(0.92,2.04) | 0.120 |  |
| **Dialysis modality** |  |  |  |  | 0.359 |
| HD | 1.75(1.18,2.59) | 0.005 | 1.67(1.11,2.51) | 0.014 |  |
| HD+HDF | 1.52(1.07,2.14) | 0.019 | 1.30(0.90,1.86) | 0.164 |  |
| HD+HP | 1.24(0.80,1.92) | 0.333 | 1.05(0.66,1.66) | 0.837 |  |
| HD+HDF+HP | 1.41(1.22,1.61) | <0.001 | 1.27(1.10,1.46) | 0.001 |  |

^*^ Adjusted for age, gender, educational level, smoking history, alcohol history, living alone, dialysis vintage, dialysis modality, dialysis access, and hemoglobin levels. Abbreviations: MetS, metabolic syndrome; MCI, mild cognitive impairment; OR, odds ratio; CI, confidence interval.
